# ‘Dependence’ as a meaningful patient‐focused outcome in Alzheimer’s disease: Qualitative concept elicitation and cognitive debriefing of the Dependence Scale in early symptomatic AD

**DOI:** 10.1002/alz.084624

**Published:** 2025-01-03

**Authors:** Alexandra Atkins, Laure Delbecque, Emma Elliott, Phoebe Heinrich, Stefan Cano, Yaakov Stern

**Affiliations:** ^1^ Eli Lilly and Company, Indianapolis, IN USA; ^2^ Modus Outcomes, Cheltenham United Kingdom; ^3^ University of Manchester, Manchester United Kingdom; ^4^ Cognitive Neuroscience Division, Columbia University, New York, NY USA

## Abstract

**Background:**

‘Dependence’ has been proposed as a measurable health outcome reflecting the overall impact of disease progression in Alzheimer’s disease (AD). However, the meaning and relevance of ‘dependence’ in early symptomatic AD (MCI and mild AD dementia) have not been previously investigated. Aims of the current research were to (1) explore the concept of ‘dependence’ from the perspective of caregivers of patients with early symptomatic AD, (2) assess the content validity of the Dependence Scale (DS; Stern, 1994), a 13‐item scale evaluating patient dependence based on a caregiver interview, and (3) explore the relevance of newly incorporated DS rater guidance clarifying potential ambiguities in item phrasing.

**Method:**

Concept elicitation (CE) and cognitive debriefing (CD) interviews were conducted with caregivers of patients with confirmed diagnoses of Mild Cognitive Impairment (MCI; n = 7) or mild AD dementia (mild AD; n = 18) recruited in the US. The DS was debriefed with 19 caregivers. CE interviews explored the concept of dependence, including its meaning and relevance. Transcribed interviews were analyzed thematically, with structured codes used for CD.

**Result:**

During CE most caregivers (n = 21) described ‘dependence’ in AD in terms of ability/lack of ability to complete tasks alone. Several (n = 16) discussed dependence in the context of concerns about leaving patients alone, citing perceived risks to physical safety or need for emotional support. Most concepts covered by the DS were discussed during interviews. During CD, some respondents (up to 42%) felt some item phrasing lacked clarity, particularly when a single response was needed to characterize functioning across multiple activities (e.g. shopping and chores); in all cases, rater guidance provides relevant clarification for use during standardized administration. Regarding content, 1 or more caregivers provided examples of patient difficulties assessed by 10 of the 13 items. Items assessing instrumental daily activities appeared most relevant, with most caregivers reporting clear examples.

**Conclusion:**

Findings support patient dependence as a meaningful concept in AD and provide evidence supporting content validity of the DS. Most DS concepts were discussed during the interviews. Rater guidance is relevant and may mitigate the impact of perceived ambiguities in item phrasing.

